# U.S. states opting out of expanded methadone take-home policies and associated mortality

**DOI:** 10.1016/j.josat.2025.209800

**Published:** 2025-09-06

**Authors:** Victor Roy, Michele J. Buonora, Cristina Murray-Krezan, Anthony Fabio, Paul J. Joudrey

**Affiliations:** a Department of Family Medicine and Community Health, University of Pennsylvania Perelman School of Medicine, Philadelphia, PA, USA; b National Clinician Scholars Program, Department of Internal Medicine, Yale School of Medicine, New Haven, CT, USA; c Veterans Affairs Connecticut Healthcare System, Yale University, West Haven, CT, USA; d Division of General Internal Medicine, Montefiore Medical Center & Albert Einstein College of Medicine, New York City, NY, USA; e Center for Biostatistics & Qualitative Methodology, Division of General Internal Medicine, University of Pittsburgh School of Medicine, Pittsburgh, PA, USA; f Department of Epidemiology, School of Public Health, University of Pittsburgh, Pittsburgh, PA, USA; g Division of General Internal Medicine, Center for Research on Health Care, University of Pittsburgh, USA

## Abstract

**Background:**

Historically, federal regulations limited take-home methadone doses largely due to concerns about methadone-related overdose. In response to the COVID-19 pandemic, an emergency federal policy in March 2020 permitted states to expand take-home methadone doses. Our objective was to utilize state-level variation in take-home expansion to compare changes in methadone related overdose death rates among states that opted into and then out of expanded take-home dosing with states that opted into and continued the policy.

**Methods:**

We used an extended two-way fixed effects difference-in-differences (DID) approach. The intervention group included states that initially opted into and then out of expanded take-home dosing, while the comparison group included states that opted into and continued the policy. Our primary outcome was the average treatment effect on the treated states (ATET) using quarterly rate of methadone-related overdose deaths per 100,000 persons from April 2020 to December 2022. Data sources included a state policy review, CDC WONDER, and U.S. Census Bureau.

**Results:**

The intervention group included three states that opted out of expanded take-home dosing, while the comparison group comprised 16 states maintaining the policy. We found no significant association between opting out of expanded take-home dosing and methadone-related overdose death rates [ATET = 0.02, 95 % CI = (−0.03, 0.47), *p* = 0.47]. Adjustments for non-methadone-related overdose variables yielded similar results.

**Conclusion:**

States who continued expanded take-home methadone dosing did not subsequently experience a detectable increase in methadone-related overdose deaths relative to states that opted out of the policy. This evidence suggests that policies expanding methadone take-homes are safe at a population level, which can inform deliberations within states that currently maintain strict restrictions.

## Introduction

Methadone treatment improves quality of life and lowers risk of fatal overdose among individuals with opioid use disorder (OUD) (Mattick et al., 2009). Limited access, engagement, and retention in methadone treatment within the US remains a challenge in responding to the ongoing overdose epidemic (Mancher & Leshner, 2019). Since the 1970s, strict regulations have governed methadone treatment for OUD, including limits on take-home methadone dosing. Prior to the COVID-19 pandemic, these regulations required patients to appear nearly daily at an opioid treatment program (OTP) for methadone dosing (Simon et al., 2022). These regulations disproportionately affect low-income patients, rural communities, and racialized minorities that are impacted by opioid use disorder and face challenges in accessing care (National Academies of Sciences, Engineering, and Medicine, 2022). However, the prevention of methadone-related overdose deaths has been a key rationale for maintaining take-home methadone restrictions, despite patient reports of reduced methadone treatment engagement and retention due to these restrictions (Jaffe and O’Keeffe, 2003; Frank et al., 2021).

In response to COVID-19, the Substance Abuse and Mental Health Services Administration (SAMHSA) issued an emergency rule in March 2020 allowing OTPs, with state concurrence, to opt into expanded take-home dosing of methadone for individuals with OUD. This policy enabled OTPs to provide up to 28 days of take-home methadone for clinically stable patients and 14 days for those deemed less stable. While most states quickly opted into the emergency rule, a minority of states held out. Of those that opted in, some states subsequently opted out and returned to pre-pandemic rules (SAMHSA, 2021). We previously found that while at least 37 states opted into expanded take-home dosing in March 2020, Ohio (March 2021), Indiana (July 2021), and Florida (May, 2022) subsequently opted out of the policy (Roy et al., 2024).

The pandemic related change in federal take-home methadone policy has been utilized as a natural experiment to evaluate the impact of take-home policies on methadone-related overdose deaths. Studies to date have primarily assessed the impact of expanded take-home dosing on methadone-related overdose deaths nationally, with the majority of studies finding no association and one study reporting an increase in methadone-related deaths following the federal emergency rule (Kleinman & Sanches, 2022; Jones et al., 2022). A limitation of these prior studies is a lack of accounting for state variation in adoption of the federal emergency rule. Inclusion of states that never opted into expanded take-home dosing or subsequently opted out of the policy may lead to biased estimates of the policy impact. Evaluation of the impact of take-home expansion among states may help inform state opioid treatment authorities (i.e., state OTP regulators) decisions related to take-home methadone policy now that federal changes were made permanent in 2024. Therefore, we utilized state-level variation in take-home expansion to compare changes in methadone related overdose death rates among states that opted into and then out of expanded take-home dosing with states that opted into and continued the policy.

## Methods

We used a difference-in-differences (DID) approach to analyze repeated cross-sectional data. DID analyses offer robustness in detecting treatment effects despite time-invariant differences between groups and common trends affecting groups over time (Callaway & Sant’Anna, 2021; Wang et al., 2024). Since the date of opt out varied among the three intervention states in our study, we used an extended two-way fixed effects model that accounts for heterogeneity in treatment effects across time and cohort (i.e., each of the three opt out states) (Callaway & Sant’Anna, 2021). In a heterogenous DID approach that uses extended two-way fixed effects, the average treatment effect on the treated (ATET) can vary among intervention states with a binary policy indicator denoting each quarter before and after a state opted out of the policy (Wang et al., 2024).

To determine a state’s quarterly take-home policy status during the study period, we used data from our prior state policy scan which included contacting state regulators to verify the time of pandemic related policy changes (Roy et al., 2024). We included states verified as adopting expanded take-home dosing in March of 2020 (SAMHSA, 2021). We excluded states with greater than two quarters of suppressed mortality data (i.e., quarters with fewer than 10 deaths). We also excluded states that did not confirm their take-home policy in March 2020 in response to our prior policy scan (Roy et al., 2024). The intervention group included states that opted out for at least two quarters in the study period after initially adopting the expanded take-home policy. The comparison group included states that continued the take-home policy. We obtained cause-specific state mortality data for all drug overdose deaths and methadone-involved deaths from Centers for Disease Control WONDER database (Centers for Disease Control and Prevention, 2024). We obtained cause-specific state mortality data for all drug overdose deaths and methadone-involved deaths from Centers for Disease Control WONDER database (i.e., drug overdose deaths included ICD-10 codes X40–X44, X60–X64, X85, and Y10–Y14; methadone-involved deaths had ICD-10 code T40.3). Quarterly methadone-involved drug overdose deaths and all other drug overdose deaths were obtained for both from April 2020 to December 2022. We obtained state demographic data from U.S. Census Bureau during this same time period).

The primary outcome was the ATET using the quarterly rate of methadone-related overdose deaths per 100,000 persons. For states with quarters of suppressed mortality data (i.e., quarters with fewer than 10 deaths), we conservatively imputed values consistent with a relative decrease in mortality in the intervention versus comparison group. We imputed 9 deaths before and 1 death after policy opt-out in the intervention group (i.e., a decline in quarterly mortality rates), and 9 deaths for all suppressed quarters in the comparison group (i.e., representing no change in mortality). Ohio’s last three quarters were excluded, because the state opted back into the expanded take home policy. Prior to our difference-in-differences analyses, we examined the trends in the quarterly rate of methadone-related overdose deaths among intervention and comparison states during the study period.

Unadjusted and adjusted models were analyzed. The adjusted model included non-methadone related overdose death rates to ensure that observed trends in mortality rate differences were not a function of states having substantially different overall overdose death rates. Analyses were done in STATA version 18. The Yale IRB determined that this research did not constitute human subjects research.

## Results

Of the 37 take-home policy adopting states in March of 2020, 18 were excluded due to greater than two suppressed quarters. The intervention group included three states: Ohio, Indiana, and Florida. The comparison group included 16 states: Arizona, California, Colorado, Georgia, Illinois, Maryland, Massachusetts, Minnesota, New Jersey, New Mexico, North Carolina, Pennsylvania, South Carolina, Texas, Virginia, Washington. Intervention group median age of the state adult population was 39.6 years, while comparison group median age was 38.9 years. Out of 33 total quarters, the intervention group had 1 suppressed quarter (3.0 %). The comparison group had 6 suppressed quarters out of a total of 176 (3.4 %). Among intervention states, at least two quarters in the study period after opting out of the expanded take-home policy: Ohio n = 4, Indiana n = 6, and Florida n = 2).

**Fig. 1.**
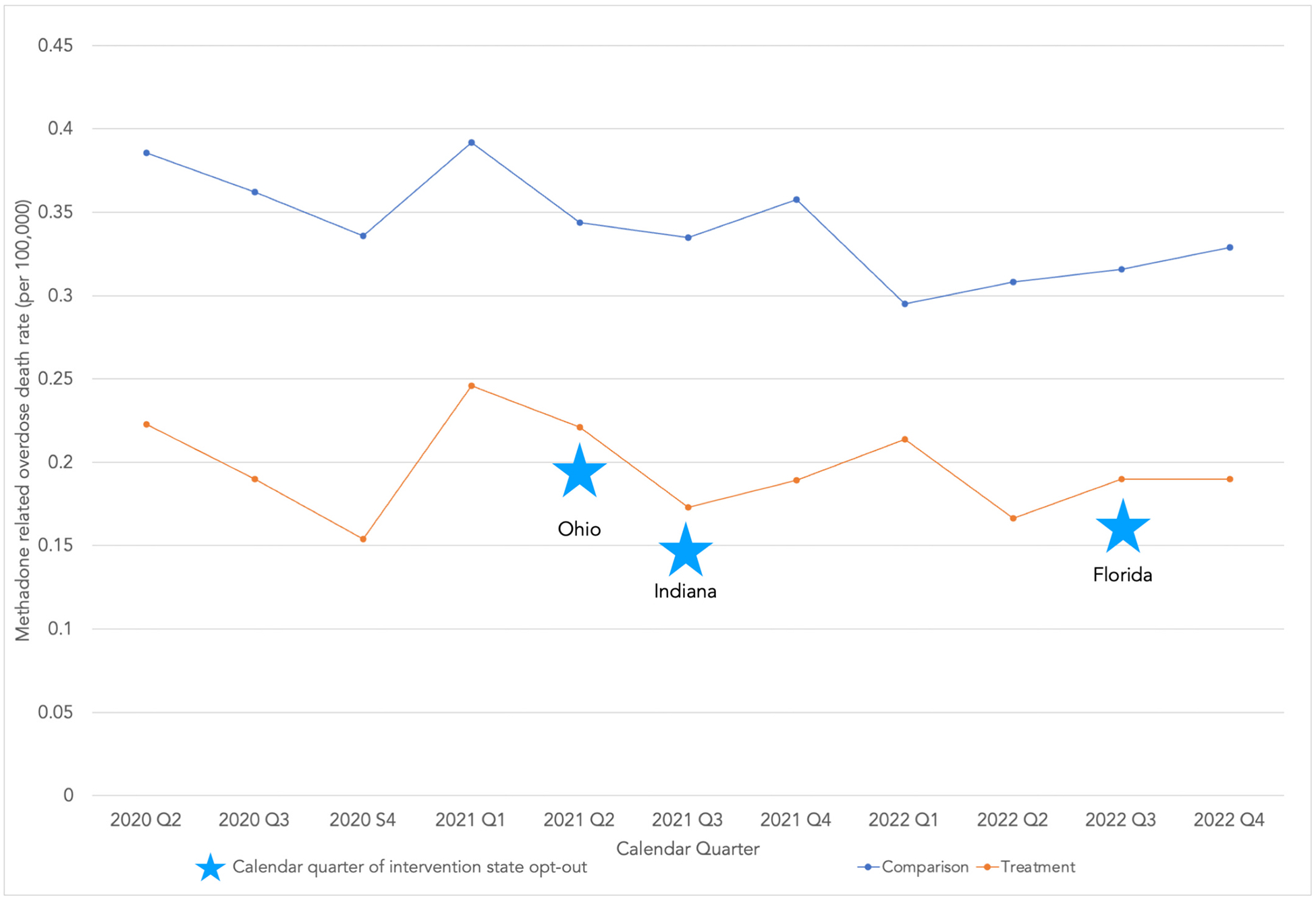
Trends in quarterly methadone-related overdose death rates. Trends in quarterly methadone-related overdose death rates (per 100,000 people) for comparison and treatment groups. Comparison group includes 16 states, and treatment group includes Ohio, Indiana and Florida, with take-home policy opt-out quarter starred for the treatment group states. Visualization of pre-treatment period indicates satisfaction of parallel trends assumption.

The trends in the quarterly rate of methadone-related overdose deaths among intervention and comparison states are reported in Fig. 1. Opting out of methadone-related treatment flexibilities did not have a detectable impact the overall methadone-related overdose death rate [ATET = 0.02, 95 % CI = (−0.03, 0.47), *p* = 0.47]. Adjusting for non-methadone related overdose death rate yielded a similar result [ATET = −0.01, 95 % CI = (−0.13, 0.11), *p* = 0.89].

**Fig. 2.**
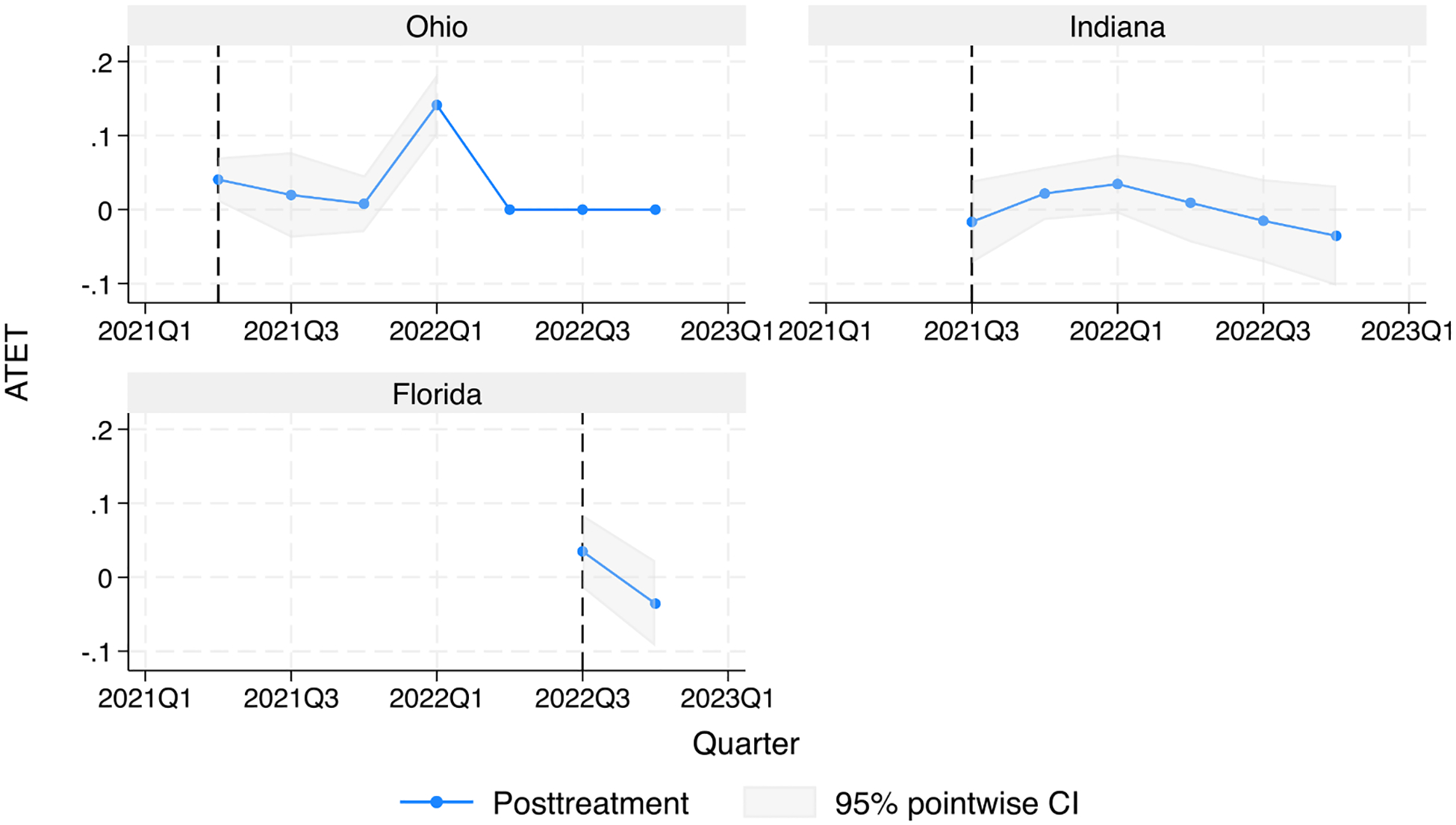
Average treatment effect on treated (intervention) states. In average treatment effect on treated (ATET), treatment effect is measured as quarterly rate of methadone-related overdose deaths per 100,000 people, depicted here in the three treated states in the post-treatment period (dotted blue line) with shaded 95 % confidence intervals.

ATET in each of the three intervention states, Ohio [ATET = 0.06, 95 % CI = (−0.09, 0.22), *p* = 0.41], Indiana [ATET = −0.03, 95 % CI = (−0.12, 0.06), *p* = 0.54], and Florida [ATET = −0.09, 95 % CI = (−0.35, 0.17), *p* = 0.48], was consistent with opting out not having a detectable impact on methadone-related overdose death (Fig. 2).

## Discussion

In this difference-in-difference analysis of repeated cross-sectional data, states who continued expanded take-home methadone dosing did not subsequently experience a detectable increase in methadone-related overdose deaths relative to states that opted out of the policy. Results among individual states in the intervention group remained consistent with this finding including after controlling for non-methadone related overdose deaths. By better accounting of state take-home policy adoption over time, our study provides further evidence that policies expanding methadone take-home dosing are not associated with population level harms. Our results are supportive of expansion of methadone take-home dosing in states that currently opt out of expansion.

Our results are consistent with a prior interrupted time series analysis of opioid overdose deaths before and after March 2020 that did not find an increase in methadone-related overdose deaths as a percentage of overall opioid-involved overdoses (Jones et al., 2022). A second national study found that methadone-related overdose deaths rose after the start of the pandemic but did not control for the rise in drug overdoses that occurred in the early months of the COVID-19 pandemic (Kleinman & Sanches, 2022). Both studies were limited by not accounting for state-level policy variation over time. A third study assessed state-level variation in immediate policy adoption in March of 2020 and did not find a relationship between expanded take-home dosing policy and methadone-related overdose deaths. However, the study did not account for multiple states subsequently opting out of the policy (Harris, 2024). An advantage of our investigation is the use of a policy scan in which we contacted state officials directly and verified policy changes over time during a period of rapid change.

Though SAMHSA updated federal methadone regulations in February 2024 to permanently expand take-home dosing, states can still choose whether to participate (SAMHSA, 2024). While a primary concern with federal policy change is the potential for increased methadone-related mortality from expanded take-home dosing, our results do not support this link. Beyond studies of potential harms from expanded take-home dosing, other analyses have pointed to the benefits of expanded take-home dosing, including patient-centered experience, lower probability of treatment discontinuation, and fewer methadone-related deaths among Black and Hispanic men (Harris et al., 2023; Krawczyk et al., 2023). These benefits are particularly critical for those, such as low-income patients and rural populations, who face transportation challenges in accessing an OTP (Joudrey et al., 2019). Our study adds to this evidence and is particularly relevant to policy deliberations within states that currently maintain strict restrictions for expanded take-home dosing for methadone despite federal policy change.

Our study has limitations. Because of overall low methadone related overdose deaths, our study may have been underpowered to detect small increases among the three intervention states. OTP and patient level studies may be required to examine small changes in benefits and harms, especially given the potential for variation in OTP-level adoption of state and federal policies. Additionally, methadone-related deaths may not be caused by methadone for OUD treatment, as methadone can also be prescribed for pain. While trends in mortality rates appear stable and comparable among intervention and comparison states, secular trends may still influence the results. Further research is needed to assess changes in state policies over time, factors influencing state-level decisions, and their impacts on treatment access and adherence.
